# Cross-Attention Transformer for Coherent Detection in Radar Under Low-SNR Conditions

**DOI:** 10.3390/s25247588

**Published:** 2025-12-14

**Authors:** Xiang Lu, Zhiwen Pan, Hengliang Zhou

**Affiliations:** 1 School of Cyber Science and Engineering, Southeast University, Nanjing 210096, China; luxiang@seu.edu.cn; 2 National Key Laboratory of Electromagnetic Effect and Security on Marine Equipment, Nanjing 210039, China; zhouhengliangbit@163.com; 3 National Mobile Communications Research Laboratory, Southeast University, Nanjing 210096, China; 4 Purple Mountain Laboratories, Nanjing 211100, China; 5 School of Information and Electronics, Beijing Institute of Technology, Beijing 100081, China

**Keywords:** coherent integration, low-RCS target detection, transformer

## Abstract

Detecting weak echoes from low-RCS targets in pulsed radar systems presents significant challenges, as conventional coherent accumulation methods require extended dwell times that reduce data rates and suffer from target-motion-induced migration. We propose RD-Transformer, an end-to-end attention-based architecture that reformulates coherent integration as a learned feature fusion problem. The framework integrates multi-pulse transpose preprocessing, dual-path self-attention encoders for transmitted and received signals, and a cross-attention decoder to extract transmit-receive interaction features. A tunable sigmoid-based gating mechanism enables flexible false alarm control during inference. Experiments on synthetic pulsed-radar data demonstrate that, under identical false alarm constraints (P_fa_ = 1 × 10^−2^ to 1 × 10^−5^) and using only 10 coherent pulses, RD-Transformer reduces the required SNR by 14–20 dB compared to optimal energy detection across Swerling I-IV target fluctuation models, validating the effectiveness of learned coherent accumulation for weak target detection.

## 1. Introduction

Detecting weak target echoes presents significant challenges in both scientific and practical domains, particularly for ultra-long-range surveillance of high-speed maneuvering objects [1], advanced-material vehicles [2], and miniaturized unmanned aerial vehicles (UAVs) [3]. These targets often exhibit markedly reduced radar cross-sections (RCSs), rendering their detection increasingly difficult. Currently, the most widely adopted strategy to improve the detection probability of such low-observable targets involves integration through coherent pulses. However, this method reduces the radar data rate and exhibits sensitivity to target-motion-induced migration across range, Doppler, and beam cells during extended accumulation periods [4,5]. Conventional techniques, including linear methods such as the Keystone transform [6,7] and Radon–Fourier transform [8], as well as non-linear approaches like the polynomial-phase transform [9], seek to mitigate motion-induced distortions by compensating for time-of-arrival variations, Doppler shifts, and higher-order phase terms. However, their effectiveness is often constrained by assumptions regarding target motion, such as constant velocity or acceleration [10]. Additionally, these algorithms require iterative parameter searches over range, velocity, or acceleration [11], relying on prior measurements that may lack precision [12]. These limitations, which restrict the achievable gains from extended accumulation, reduce the robustness of the methods and decrease target detection efficiency.

Transformers offer advantages such as better capability to capture long-range dependencies and global context through their self-attention mechanisms [13], and improved scalability with larger datasets [14]. Therefore, Transformer-based radar object detection holds significant promise. Recent architectures such as RadarFormer [15] and T-RODNet [16] combine Transformer and CNN modules to achieve target detection. However, these designs have demonstrated almost exclusively on Frequency Modulated Continuous Wave Radar, and comparable approaches for coherent accumulation in pulse-radar systems remain unexplored. Moreover, the CNN-based dimension-mapping required to fuse two elaborate models incurs a substantial computational overhead, and the use of fixed false alarm control during training precludes flexible adjustment of the false alarm level during inference.

Motivated by recent success of Transformers in radar object detection [15,16] and time-series forecasting [17], this paper introduces a hybrid framework designed to address multi-pulse coherent accumulation for pulsed-radar target detection. It integrates a specialized data preprocessing module for complex I/Q sampling, a cross-attention mechanism to extract transmit–receive interaction features from echoes, and a robust training strategy that combines noise injection with early stopping. The main contributions of this paper are as follows:

(1) By integrating self-attention and cross-attention mechanisms into the pulsed-radar coherent-accumulation process, the proposed network reduces the required SNR by 14–20 dB compared to optimal coherent energy detection methods under the same false alarm constraints (P_fa_ = 1 × 10^−2^ to 1 × 10^−5^) across all Swerling I–IV fluctuation models, relative to optimal coherent energy detection.
(2) By significantly reducing the required accumulation pulses, our approach not only enhances detection efficiency but also enables faster and more comprehensive updates of the radar’s coverage, ensuring timely responsiveness in dynamic environments.
(3) By leveraging high-dimensional vector embeddings and encoder–decoder network architectures, the RD-Transformer provides an end-to-end solution for radar signal integration and target detection, in which the known transmit waveform is treated as an explicit reference branch that interacts with the received echoes, analogous to a learned matched filter that replaces the fixed filter and covers the entire coherent accumulation process.

The remainder of this paper is organized as follows. Section 2 reviews conventional radar detection methods and recent deep-learning- and Transformer-based approaches for radar target detection. Section 3 introduces the signal model and problem formulation for coherent pulse radar. Section 4 details the proposed RD-Transformer workflow, including sliding-window preprocessing of I/Q echoes, transmit/receive embedding with positional encoding, dual-path encoders, cross-attention-based transmit-receive feature fusion, and the k-sigmoid false alarm control mechanism. Section 5 presents the data generation strategy, experimental setup, comparative results, ablation studies, and inference-speed evaluation. Section 6 concludes the paper and discusses future research directions.

## 2. Related Work

### 2.1. Traditional Radar Detection Methods

Pulse-Doppler radar target detection, as a core technology in modern radar systems, relies on effective joint extraction of range and Doppler two-dimensional information from radar echoes to achieve high-resolution ranging, velocity measurement, and clutter suppression for moving targets. Traditional target detection methods are primarily based on statistical characteristics, including Likelihood Ratio Test (LRT), Track Before Detect (TBD), and Constant False Alarm Rate (CFAR) techniques. These conventional approaches, built upon CFAR detection, matched filtering, and Doppler spectrum analysis combined with target detection and tracking algorithms, still face performance bottlenecks in complex backgrounds and multi-target environments [18].

The rich information contained in radar echo signals provides favorable conditions for feature-based detection techniques. Traditional statistical-based detection methods face increasing difficulty in statistical modeling due to the complexity of echo signals, making it challenging to effectively extract useful features from echo information and differentiate between target and interference characteristics. Existing research has explored various radar echo features including fractal characteristics, chaotic dynamics, time-frequency domain features, polarization features, and multi-feature fusion for radar target detection.

#### 2.1.1. Fractal Features

Fractal theory has been extensively applied to target detection. Ward et al. [19] pioneered the application of fractal dimension analysis to sea clutter modeling, demonstrating that sea clutter exhibits fractal properties over certain time and spatial scales. Subsequently, Hu et al. [20] developed multifractal-based detection methods using the Hurst exponent to characterize sea clutter temporal correlation, achieving improved detection performance in non-Gaussian clutter environments. Recent advances have also explored frequency-domain fractal characteristics and autoregressive (AR) spectrum fractal properties, demonstrating consistent detection performance improvements [21].

#### 2.1.2. Chaotic Features

In 1995, Haykin et al. [21] first revealed that radar sea clutter exhibits chaotic dynamics, utilizing correlation dimension, Kolmogorov entropy, and Lyapunov exponents to construct prediction models for target detection. Leung and Lo [22] extended this work by developing neural network-based chaotic prediction methods that reconstruct phase space from radar measurements. Haykin [23] further demonstrated the feasibility of nonlinear dynamics-based detection algorithms using real IPIX radar data with various prediction models.

#### 2.1.3. Time-Frequency Domain Features

Time-frequency analysis provides powerful tools for characterizing non-stationary radar signals. Chen [24] extensively studied micro-Doppler signatures as time-varying features for target classification, while [25] provided a comprehensive framework for time-frequency distributions applicable to radar signal analysis. Stankovic [26] proposed adaptive time-frequency representations for enhanced radar signal processing. In the context of Doppler spectrum features, Shui et al. [27] developed entropy-based features derived from Doppler spectrum characteristics, proposing detection algorithms based on Doppler waveform entropy.

#### 2.1.4. Multi-Feature Fusion Detection

Multi-feature fusion has emerged as a promising approach to leverage complementary information from different domains. Shui et al. [27] pioneered three-feature fusion by combining time-domain relative mean amplitude with frequency-domain relative Doppler peak height and Doppler spectrum entropy, developing effective fusion-based detection algorithms. Park et al. [28] proposed joint detection methods using average power and Doppler spectrum entropy with non-additive fusion models. Recent trends show increasing feature dimensions in multi-domain fusion. More recently, ref. [29] explored high-dimensional feature spaces by extracting dimensional features from time, frequency, wavelet, and bispectrum domains, utilizing stacked autoencoders for feature fusion to construct advanced interference recognition and detection models.

Despite the progress achieved with hand-crafted features, traditional statistical-based detection methods exhibit inherent limitations that constrain their performance in complex and dynamic radar environments.

First, hand-crafted features are typically designed based on specific statistical models or physical assumptions about the target characteristics [30,31]. This model-dependent nature makes them vulnerable to deviations from assumed statistical distributions, variations in scattering mechanisms, or changes in observation conditions such as environmental non-stationarity, multipath effects, target velocity/acceleration variations, and Doppler ambiguity [32].

Second, the limited dimensionality and expressive capacity of hand-crafted features pose fundamental challenges. Traditional approaches typically employ a small number of manually selected features (often fewer than 10 dimensions), which may be insufficient to capture the high-order, cross-domain, and highly nonlinear relationships inherent in radar echo signals [33,34]. As noted in the context of machine learning, hand-engineered features often fail to capture the full complexity of data distributions, especially when the underlying structure is intricate and multi-scale. This limitation becomes particularly acute in low signal-to-noise ratio (SNR) scenarios or complex backgrounds where subtle discriminative information is embedded in high-dimensional feature spaces that hand-crafted features cannot adequately represent [35].

### 2.2. Deep Learning-Based Radar Target Detection Methods

In recent years, deep learning has revolutionized radar target detection by enabling automatic feature learning and robust pattern recognition from complex radar data. Unlike traditional hand-crafted feature-based methods, deep neural networks can learn hierarchical representations directly from raw or minimally preprocessed radar signals, achieving superior performance in challenging environments [34]. Based on the preprocessing strategies and data representations employed, deep learning-based radar target detection can be categorized into three main approaches: one-dimensional (1D) time-series signal detection, Range-Doppler (R-D) map-based detection, and micro-Doppler signature-based detection.

#### 2.2.1. One-Dimensional Time-Series Signal Detection

Radar echo signals in the time domain can be treated as sequential data, where temporal dependencies carry critical information for target discrimination. Recurrent Neural Networks (RNNs) and their variants, particularly Long Short-Term Memory (LSTM) networks and Gated Recurrent Units (GRUs), have demonstrated exceptional capability in modeling temporal patterns in radar sequences [36,37]. Kim and Moon [38] pioneered the application of LSTM networks for human activity classification using micro-Doppler radar signatures, achieving over 90% accuracy across multiple activity classes. Their work demonstrated that LSTM networks can effectively capture the temporal evolution of radar returns, outperforming traditional feature-based classifiers.

More recently, Temporal Convolutional Networks (TCNs) have emerged as powerful alternatives to RNNs for processing radar time-series data [39]. TCNs leverage dilated causal convolutions to capture long-range temporal dependencies while maintaining parallel processing efficiency. Angelov et al. [40] applied deep learning to radar-based human activity recognition, demonstrating that 1D convolutional architectures can achieve real-time performance with accuracy exceeding 95%. However, 1D signal processing approaches typically utilize only amplitude or phase information from a single range bin or Doppler cell, potentially limiting their ability to exploit the full spatial-spectral structure of radar returns [41].

#### 2.2.2. Range-Doppler Map Based Detection

Range-Doppler (R-D) maps, obtained by applying Fast Fourier Transform (FFT) along both fast-time (range) and slow-time (Doppler) dimensions, provide a two-dimensional representation of target energy distribution that is naturally suited for image-based deep learning methods [42]. Convolutional Neural Networks (CNNs) have been extensively applied to R-D map analysis, leveraging spatial convolutions to extract discriminative features from these spectrograms.

Chen et al. [43] developed a CNN-based automatic target recognition system for Synthetic Aperture Radar (SAR) images, achieving state-of-the-art classification performance on the MSTAR dataset with over 99% accuracy. Their work demonstrated that deep CNNs can learn robust features invariant to target aspect angle, depression angle, and configuration variations. For small target detection in sea clutter, Xu et al. [35] proposed a CNN-based detector that processes R-D maps and significantly outperforms traditional CFAR methods, reducing false alarm rates by over 40% while maintaining high detection probabilities.

Object detection frameworks originally developed for computer vision have been successfully adapted to radar R-D map analysis. The YOLO (You Only Look Once) family of single-stage detectors has gained popularity due to its real-time processing capability [44]. Ren et al. [45] applied Faster R-CNN to object detection tasks, demonstrating significant improvements in both speed and accuracy over previous region-based methods. The Faster R-CNN framework, with its Region Proposal Network (RPN), has proven particularly effective for detecting multiple targets in cluttered radar scenes, achieving detection rates above 90% while maintaining low false alarm rates.

Feature Pyramid Networks (FPNs) address the challenge of detecting targets at multiple scales by constructing multi-scale feature hierarchies [46]. Lin et al. [46] demonstrated that FPNs can detect objects ranging from small to large sizes with consistent performance, which is particularly relevant for radar systems detecting targets at varying distances. More recently, transformer-based architectures have been explored for radar target detection [17]. The self-attention mechanism in transformers enables modeling of long-range dependencies in R-D maps, potentially capturing subtle target signatures that CNNs might miss. Carion et al. [47] introduced DETR (DEtection TRansformer), which reformulates object detection as a direct set prediction problem, eliminating the need for hand-designed components like non-maximum suppression.

#### 2.2.3. Micro-Doppler Signature-Based Detection

Micro-Doppler signatures, arising from micro-motions of target components (e.g., rotating blades, swinging limbs, or vibrating surfaces), provide distinctive temporal-frequency patterns that enable fine-grained target classification [26]. Time-frequency analysis tools such as Short-Time Fourier Transform (STFT), Wigner-Ville Distribution (WVD), and Continuous Wavelet Transform (CWT) transform 1D radar returns into 2D time-frequency spectrograms that reveal micro-motion characteristics [27].

Deep learning has proven highly effective for micro-Doppler-based target recognition. Kim and Moon [38] applied CNN architectures to micro-Doppler spectrograms for human activity classification, demonstrating that CNNs can automatically learn discriminative features from time-frequency representations without manual feature engineering. Their experiments showed that deep CNNs outperform traditional machine learning methods (e.g., Support Vector Machines with hand-crafted features) by significant margins, achieving accuracies above 95%.

For UAV detection and classification, which has become increasingly important for airspace security, Ritchie et al. [48] demonstrated that CNN-based analysis of micro-Doppler signatures can reliably distinguish between different UAV types based on their unique rotor blade flash patterns. The periodic modulation caused by rotating propellers creates distinctive patterns in time-frequency representations that deep networks can learn to recognize. Jokanović et al. [49] proposed a CNN-based approach for radar fall detection in eldercare applications, achieving over 96% classification accuracy on micro-Doppler spectrograms. Their work highlighted the importance of data augmentation and transfer learning when training data is limited, a common challenge in radar applications.

Recent advances have explored hybrid architectures combining CNNs for spatial feature extraction with RNNs for temporal modeling. Du et al. [50] proposed a CNN-LSTM architecture for human motion classification using micro-Doppler signatures, where CNN layers extract spatial features from spectrograms and LSTM layers model temporal evolution across consecutive time frames. This hybrid approach achieved superior performance compared to pure CNN or RNN architectures, particularly for activities with complex temporal dynamics.

Transfer learning has also been successfully applied to micro-Doppler analysis. Malmgren-Hansen et al. [51] demonstrated that CNNs pre-trained on large-scale image datasets (e.g., ImageNet) can be fine-tuned for radar target recognition tasks, significantly reducing training time and improving generalization performance when labeled radar data is scarce. This approach leverages the hierarchical feature representations learned from natural images, which often transfer well to other visual recognition tasks including radar spectrograms.

### 2.3. Transformer-Based Radar Target Detection Methods

In recent years, the Transformer architecture, originally designed for natural language processing, has been increasingly adapted to radar target detection tasks, demonstrating remarkable capabilities in modeling long-range dependencies and capturing global contextual information from radar signals. Unlike traditional convolutional neural networks (CNNs) that rely on local receptive fields, Transformers leverage self-attention mechanisms to establish direct connections between all positions in the input sequence, enabling more effective feature extraction from complex radar returns [17]. This paradigm shift has led to diverse approaches in applying Transformers to radar target detection, which can be categorized based on their training strategies and architectural characteristics.

The adaptation of Transformer architectures to radar target detection follows three primary methodologies that represent different design philosophies and application scenarios. First, end-to-end Transformer models [52] are trained from scratch on specific radar detection tasks, directly learning task-specific feature representations from raw or minimally preprocessed radar data without leveraging external knowledge sources. Second, pre-trained Transformer models [53] employ transfer learning strategies, where models initially trained on large-scale datasets (either from radar or related domains) are fine-tuned for specific detection tasks, capitalizing on learned representations to improve performance particularly when labeled radar data is limited. Third, multi-channel temporal Transformer models are specifically designed to exploit the unique characteristics of radar signals, including multi-channel structure (e.g., I/Q components, multiple polarizations) and temporal dependencies across pulse sequences, often combining Transformers with recurrent architectures to capture both local temporal dynamics and global contextual relationships [54]. These three approaches are complementary rather than mutually exclusive, and their integration represents promising directions for advancing radar target detection capabilities.

The aforementioned methods, whether end-to-end Transformers or hybrid architectures combining Transformers with CNNs or RNNs, have demonstrated superior performance across multiple public and proprietary datasets. Results consistently show that Transformer architectures can effectively capture global features in radar signals and significantly outperform traditional methods across various tasks. However, several critical challenges remain in applying Transformers to radar target detection:

First, existing research predominantly focuses on millimeter-wave radar, continuous-wave radar, and automotive radar applications. For coherent pulse radar systems commonly employed in air surveillance and maritime monitoring, how to effectively leverage Transformer architectures to implement efficient coherent integration and target detection from transmitted and received pulse sequences remains an open research question requiring specialized investigation [42].

Second, current approaches often convert input data into simple time-sampled sequences for processing, which is computationally inefficient and resource-intensive, particularly for high pulse repetition frequency (PRF) radar systems generating massive data volumes. Developing effective encoding schemes that facilitate efficient feature extraction while preserving essential signal characteristics represents a fundamental challenge [55].

Third, controlling and adjusting detection probability and false alarm rate dynamically remains difficult with end-to-end learning approaches. Unlike traditional CFAR (Constant False Alarm Rate) detectors that allow explicit threshold adjustment to achieve desired operating points on the receiver operating characteristic (ROC) curve, end-to-end trained neural networks typically produce fixed detection probabilities and corresponding false alarm rates determined during training, lacking mechanisms for post-deployment adaptation to varying operational requirements or environmental conditions [18]. Addressing this limitation is essential for practical deployment in operational radar systems where mission requirements and electromagnetic environments may change dynamically.

Beyond classical energy detection, a variety of advanced weak-target detectors have been proposed, including long-time coherent integration schemes based on Keystone and Radon-Fourier transforms, Lv’s distribution, and related motion-compensation techniques [5,6,7,8,9,56,57,58,59,60,61], model-based detectors for sea clutter with K-distributed or compound-Gaussian statistics [31,62], and multi-feature or learning-based CFAR variants [27,28,35]. These approaches can yield substantial gains in specific scenarios, but they typically rely on precise motion-parameter search or strong assumptions about clutter statistics, and they are mostly designed for narrowband, sea-clutter, or range-Doppler-map processing rather than for pulse-level coherent integration with only a few pulses under extremely low SNR. In this context, the optimal coherent energy detector implemented with full pulse compression and range-Doppler accumulation remains a widely accepted performance upper bound for conventional pulsed-radar processing chains.

## 3. Signal Model and Problem Formulation

Consider a coherent radar system operating in pulsed mode. Let the complex baseband echo data after analog-to-digital conversion be organized into an *n × m* matrix $P_{echo}\in C^{n\times m}$, where *n* denotes the number of range bins (fast-time dimension) and *m* represents the number of coherent pulses (slow-time dimension). The matrix structure is expressed as

(1) $$P_{echo}=\left[\begin{array}{c} e_{1}\\ e_{2}\\ \begin{array}{c} \ldots \\ e_{n} \end{array} \end{array}\right]=\left[\begin{array}{cccc} e_{11} & e_{12} & \ldots & e_{1m}\\ e_{21} & e_{22} & \ldots & e_{2m}\\ \vdots & \vdots & \ddots & \vdots \\ e_{n1} & e_{n2} & \ldots & e_{nm} \end{array}\right]=\left[\begin{array}{c} e_{1}^{T}\\ e_{2}^{T}\\ \begin{array}{c} \vdots \\ e_{n}^{T} \end{array} \end{array}\right],$$

where $e_{i,j}\in C^{n\times m}$ represents the complex-valued echo sample (detailed in Equation (2)) at the *i*-th range bin and *j*-th pulse, and $e_{i}={\left[e_{i,1},e_{i,2},\ldots ,e_{i,m}\right]}^{T}\in C^{m}$ denotes the temporal pulse sequence at the *i*-th range cell.

For a given range cell, the received echo $e_{i}$ can be decomposed into its in-phase (I) and quadrature (Q) components. Each pulse sample $e_{i,j}$ is represented as

(2) $$e_{i,j}=I_{i,j}+jQ_{i,j},$$

where $j=\sqrt{-1}$, and $I_{ij}$, $Q_{ij}\in R$ are the in-phase and quadrature components, respectively [63]. In practice, the received echo comprises two primary components: (i) the reflected signal from targets denoted as $e_{reflect,i,j}$, and (ii) the thermal noise and interference, denoted as $e_{noise,i,j}$. Thus, (2) can be expanded as

(3) $$e_{i,j}=e_{reflect,i,j}+e_{noise,i,j},$$

Throughout this work, we adopt the compact notation $e_{i}(m)\equiv e_{i,m}$ to emphasize the temporal evolution of echoes within a specific range cell, where *m* indexes the slow-time (pulse) dimension.

The radar transmits a linear frequency modulation (LFM) chirp signal, which is widely employed in coherent radar systems due to its favorable range resolution and pulse compression properties [4,58]. The complex baseband representation of the transmitted LFM signal is given by

(4) $$s_{t}\left(t\right)=rect\left(\frac{t}{T_{p}}\right)exp\left(j2\pi f_{c}t+j\pi \gamma t^{2}\right),$$

where $rect\left(.\right)$ is the rectangular window function defined as

(5) $$rect\left(u\right)=\left\{\begin{array}{c} 1,\left|u\right|\leq 1/2\\ 0,\left|u\right|>1/2 \end{array}\right..$$

$T_{p}$ is the pulse width, γ represents the chirp rate (frequency modulation rate), $f_{c}$ denotes the carrier frequency, and *t* is the fast-time variable.

For a point target with radar cross-section (RCS) σ, the received echo in baseband form after dechirping can be expressed as [4,58]

(6) $$s\left(t,t_{m}\right)=\sigma rect\left[\frac{t-2R\left(t_{m}\right)/c}{T_{p}}\right]exp\left[j\pi \gamma {\left(t-\frac{2R\left(t_{m}\right)}{c}\right)}^{2}\right]\times exp\left[-j\frac{4\pi }{\lambda }R\left(t_{m}\right)\right],$$

where $t_{m}$ denotes the slow time (pulse repetition time index), c is the speed of light, λ is the radar wavelength, and $R\left(t_{m}\right)$ represents the instantaneous slant range between the radar and the target at slow time $t_{m}$.

Unlike conventional approaches that model targets with constant velocity [64,65,66], constant acceleration [59,67], or jerk motion [8], this work adopts a generalized polynomial motion model to characterize high-maneuverability targets (HMTs) with arbitrary-order kinematics. The instantaneous range $R\left(t_{m}\right)$ is modeled as a *z*-th order polynomial [58,60]:

(7) $$R\left(t_{m}\right)=R_{0}+\sum_{i}^{z}\alpha_{i}t_{m}^{i}R_{0}+\alpha_{1}t_{m}+\alpha_{2}t_{m}^{2}+\ldots +\alpha_{z}t_{m}^{z},$$

where $R_{0}$ is the initial slant range at $t_{m}$, *z* is the maximum motion order, and ${\left\{\left.a_{i}\right\}\right.}_{i=1}^{z}$ are the motion coefficients corresponding to the *i*-th order radial motion parameter. Specifically, $\alpha_{1}$ represents the radial velocity, $\alpha_{2}$ represents half the radial acceleration, $\alpha_{3}$ corresponds to one-sixth of the radial jerk, and so forth. This unified representation enables consistent treatment of diverse target dynamics.

The time-varying range $R\left(t_{m}\right)$ in (7) induces corresponding Doppler and micro-Doppler modulations in the received signal, which serve as discriminative signatures for target detection. Instead of relying on explicit parameter estimation and threshold-based detection—approaches that often suffer from performance degradation in low signal-to-noise ratio (SNR) regimes and complex environments [4]—we formulate detection as a binary classification problem.

For each range cell, we define the classification task as determining whether a target is present or absent. Let $\xi \in \left\{0,1\right\}$ denote the binary label, where ξ=1 indicates target presence and *ξ* = 0 indicates target absence. The detection problem is then formulated as

(8) $$\hat{\xi }=F\left(P_{echo};\Theta \right),$$

where $F\left(.;\Theta \right)$ represents a deep neural network (DNN) with learnable parameters Θ, and $\hat{\xi }$ is the predicted label. The function *F*(*·*; Θ) encompasses multiple nonlinear transformations that enable the network to learn hierarchical feature representations directly from the raw range-Doppler data.

## 4. Method

**Figure 1:**
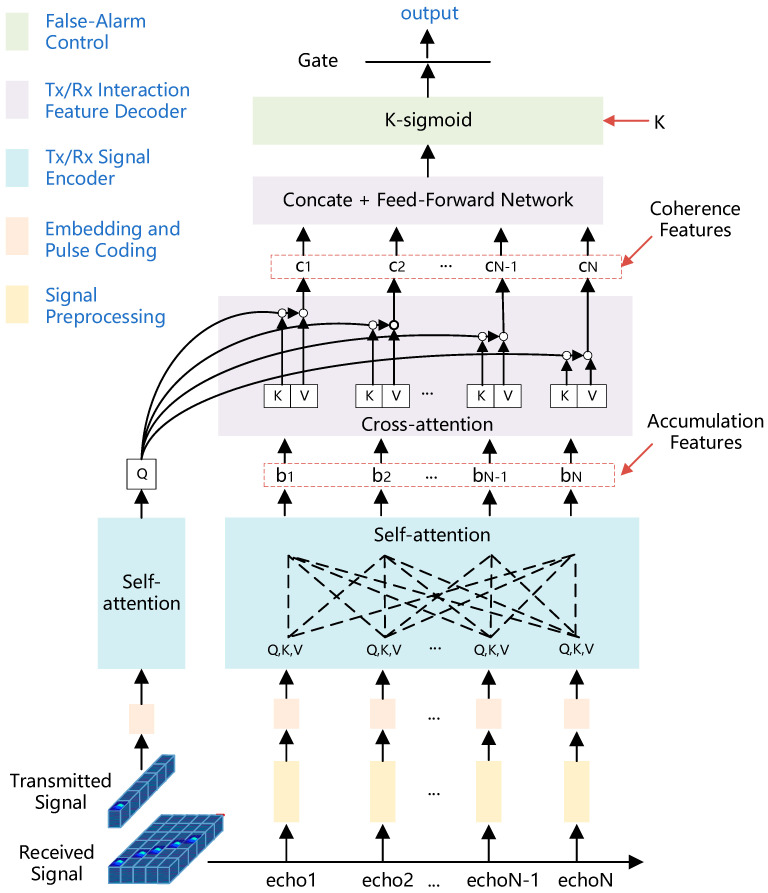
RD-Transformer architecture.

We propose the RD-Transformer, as illustrated in Figure 1, an end-to-end attention-based architecture that reformulates coherent accumulation as an adaptive feature fusion problem. Unlike conventional FFT-based methods that assume stationary targets, our cross-attention mechanism learns to dynamically weight and align multi-pulse echoes, implicitly compensating for target-induced phase variations across range-Doppler cells. This data-driven approach enables robust detection under motion-induced migration without explicit parameter estimation. First, a data-preprocessing module reduces token count to lower computational load; embedding layers and positional encodings then project transmit and receive pulse sequences into a compact, position-aware feature space. Dual encoders apply self-attention to coherently accumulate receive pulses and to abstract the transmitted signal. A cross-attention decoder fuses these representations, capturing interdependencies between transmit and receive features for multi-pulse coherent accumulation. Finally, a learnable k-sigmoid function—whose steepness is directly controlled by coefficient k—provides tunable false alarm control at inference.

### 4.1. Radar Signal Preprocessing

For radar echo signals, each sampling moment contains quadrature (Q) and in-phase (I) components, as well as the sampling time (T). A single pulse’s fast-time sampling yields PRI × Fs data points, and N coherent pulses increase the total number of points by N-fold. Directly treating each sampling point as a token for input into a transformer results in prohibitively long sequences, leading to exponential growth in computational and memory demands, thereby limiting the efficiency of model training and inference.

To address this, we propose two preprocessing strategies:

(1) Patch-based processing

**Figure 2:**
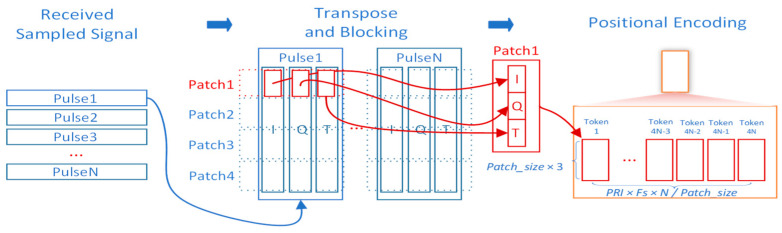
Patch-based processing inspired by ViT and ITRANSFORMER.

Figure 2 presents the patch-based processing strategy. Fast-time data are divided into patches, and each patch, comprising Patch_size fast-time samples, is reshaped into a Patch_size dimensional vector. Data from an entire coherent processing interval (CPI), spanning fast and slow time dimensions, are processed into patches and fed into the model for target detection. Compared to treating all fast-time samples as tokens, this approach significantly reduces the token count, improving training efficiency. While this method effectively determines target presence within a region, estimating precise target locations requires additional feature flattening and mapping.

From the perspective of coherent integration, each patch is still formed from the full set of coherent pulses within a CPI. The time dimension (i.e., the pulse index) is preserved when constructing the patch sequence, so the phase/time coherence across pulses that is needed for coherent integration is not destroyed by the patching or reshaping operations. In implementation, the long fast-time sampling sequence is first transformed into a higher-dimensional feature representation and then reshaped into multiple patches; the subsequent mappings operate on all patch elements and can implement point-wise accumulation as a special case. As a result, fine-grained phase and timing information remains available for the network to exploit, even for low-RCS or fast-moving targets.

The choice of Patch_size controls the trade-off between token length and feature depth. In our design, Patch_size can in principle vary from 1 (no aggregation along fast time, maximal token length) to the total number of fast-time samples within the CPI (one token per CPI, maximal feature depth). Very small Patch_size values increase the number of tokens and lead to quadratic growth in attention computation and memory consumption, whereas excessively large Patch_size values concentrate too many samples into a single high-dimensional token, which may make optimization difficult and reduce flexibility in modeling local range structure. In practice, Patch_size is chosen to balance detection performance and computational cost under available hardware resources.

(2) Sliding-Window processing

**Figure 3:**
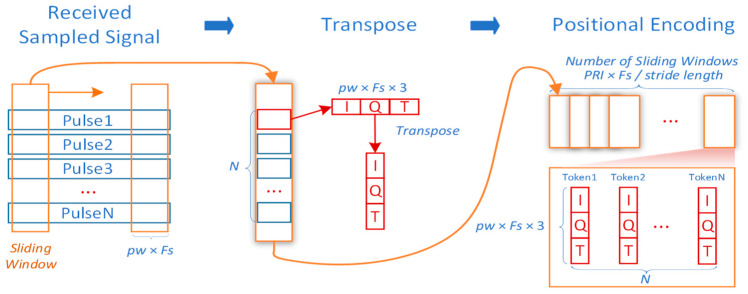
Sliding-Window processing inspired by traditional radar.

Figure 3 presents the sliding-Window processing strategy. A sliding window of length PW × Fs is applied along the fast-time dimension. For each pulse, fixed-length fast-time data within the window are extracted across all pulses to form a sub-pulse sequence. This sequence retains the original number of pulses but with reduced fast-time samples (PW × Fs). The sampled I/Q components and timestamps are flattened, concatenated, and transformed into d_model_ dimensional vectors via embedding. N coherent pulses yield N tokens. Here, each token is formed from a three-channel vector [I(t), Q(t), T(t)], where T(t) denotes the fast-time sampling index within the pulse. The inclusion of T(t) provides an explicit intra-pulse temporal coordinate for every sample inside the sliding window, enabling the model to distinguish where a given I/Q sample lies within the pulse duration and to better capture Doppler- and phase-evolution patterns that manifest as structured changes along fast time. When only local windows are observed, this additional temporal cue compensates for the loss of global fast-time context and makes the sliding-window representation sensitive to physically meaningful time-ordering information. The received signals, limited to the pulse duration PW, undergoes the same embedding and is fed into the model for detection. By sliding the window across the fast-time dimension, multiple sub-pulse pairs are generated, and the model performs detection iteratively to provide target presence markers corresponding to range cells. This approach not only reduces the dimensions of inverse-transformed data and token counts but also enables effective target range estimation.

All evaluation results presented in this study are based on the sliding-window preprocessing approach, demonstrating its efficacy for rapid regional target detection and localization.

### 4.2. Embedding and Pulse Positional Encoding

The raw transmit and receive signals are first mapped into a shared, lower-dimensional feature space via learnable linear projections. Specifically, the transmit embedding is computed as

(9) $$S_{a}=W_{a}A^{\prime }+b_{a}\in R^{1\times B\times d_{model}},$$

and the receive embedding as

(10) $$S_{b}=W_{b}B^{\prime }+b_{b}\in R^{N_{p}\times B\times d_{model}},$$

where $d_{model}=2048$, B denotes batch size, $N_{p}$ is the number of coherent pulses, and $W_{a}$, $W_{b}$ are trainable parameters. Before forming $A^{\prime }$ and $B^{\prime }$, all three input channels I, Q, and T within each sliding window are linearly normalized (zero mean and unit variance computed over the training set). This normalization prevents very small-magnitude echoes from being numerically drowned by floating-point precision limits, keeps the activations away from saturated linear regimes, and improves gradient propagation during training. To preserve the intrinsic ordering of the receive pulse sequence, we add sinusoidal positional encodings to

(11) $${PE}_{\left(pos,2i\right)}=sin\left(\frac{pos}{{10,000}^{2i/d_{model}}}\right),$$

(12) $${PE}_{\left(pos,2i+1\right)}=cos\left(\frac{pos}{{10,000}^{2i/d_{model}}}\right).$$

These encodings inject explicit pulse-index information, ensuring that parallel attention operations maintain awareness of the temporal relationships among pulses.

### 4.3. Encoding of Transmit and Receive Signals

To preserve the distinct characteristics of the transmit and receive signals, we adopt a dual-path transformer encoder, in which the embedded transmit and receive signals are processed by two parallel encoders. Unlike a shared encoder that maps both the noise-free transmit signal and the noisy receive signal through the same transformation, the proposed design enables dedicated representation learning for each signal type. The transmit path performs deep encoding of the ideal reference signal, generating Query (Q), Key (K), and Value (V) representations for subsequent attention-based detection. Meanwhile, the receive path accumulates features across multiple coherent echo pulses using multi-head self-attention, effectively capturing long-range dependencies and inter-pulse correlations. This process yields N aggregated features—where N corresponds to the number of pulses—each incorporating information from the full temporal context. From a radar signal-processing perspective, the Tx encoder plays a role similar to the reference channel in classical matched filtering, while the Rx encoder aggregates noisy echoes over multiple pulses. By feeding both encoded Tx and Rx features into the subsequent cross-attention decoder, the network learns a generalized, data-driven matched filter and coherent integrator that explicitly exploits Tx-Rx coherence instead of relying solely on the received signal. This explicit use of the transmit waveform as a reference preserves the physical interpretability of the detector and enables the model to compensate for motion-induced phase migration across pulses that fixed, Rx-only processing cannot handle effectively.

### 4.4. Extraction of Transmit–Receive Interaction Features

To capture the deep relationship between the receive and the transmitted signal, we employ a transformer decoder to compute interaction attention in parallel between each received-pulse feature and the transmitted-signal feature, yielding a set of coherence features. These features are then flattened and passed through a fully connected neural network to aggregate the pulse–transmit coherence features into logit, effectively representing the accumulated interaction information across multiple pulses and the transmitted signal. In contrast to conventional detectors that operate only on processed received signals (e.g., after a fixed matched filter and coherent integration), RD-Transformer keeps the transmit waveform in the loop so that the decoder attends directly to a clean Tx reference and implements a learnable Tx-Rx correlation stage that generalizes classical matched filtering to the full coherent accumulation process.

### 4.5. False Alarm Control Mechanism

To map network logits into detection probabilities and make final binary decisions, we employ a parameterized sigmoid function

(13) $$P_{detect}=\sigma \left(ky_{logit}\right)=\frac{1}{1+exp(-ky_{logit})},$$

where the scalar k (e.g., k=1/1.6) modulates the sigmoid’s slope: larger values of k yield a steeper transition around zero, enabling finer control over the resulting probability distribution. A detection is declared whenever exceeds an adjustable threshold θ:

(14) $$Decision=\left\{\begin{array}{c} 1,\;P_{detect}>\theta ,\\ 0,\;otherwise. \end{array}\right.$$

By jointly tuning k and θ, the system adaptively balances detection probability against false alarm rate under varying signal-to-noise conditions, thus providing robust performance across diverse operating regimes. In practice, we treat the detection gate θ as a coarse operating-point control and the slope parameter k as a finer shaping parameter for the output probability distribution. Starting from a single trained network, we first sweep θ on a validation set to satisfy a desired false alarm constraint; when a lower false alarm rate is required, θ is increased to impose a more stringent decision gate. If θ approaches the extremes of [0, 1] or the logits become too peaky, k is gently reduced to flatten the sigmoid mapping and θ is increased accordingly, jointly moving the detector toward more conservative decisions. This two-parameter calibration enables us to realize different trade-offs between $P_{d}$ and $P_{f}$ with one trained model, instead of retraining separate networks for each operating point.

### 4.6. Optimization Details

The training process integrates noise injection and early stopping to enhance model performance and robustness. During the initial training phase, substantial noise is introduced to encourage the model to escape local minima, promoting stochasticity and enabling better exploration of the parameter space. As training progresses, the noise level is gradually reduced, allowing the model to stabilize and converge to an optimal solution. This approach improves the model’s ability to explore complex parameter spaces, enhances generalization, and mitigates overfitting risks. Early stopping is implemented with a tolerance of 5 epochs, and the signal-to-noise ratio (SNR) increases by 0.1 dB per step.

The training and validation datasets are generated using either a point target model or an RTX ray-tracing model, with 1 million training samples per SNR scenario and 2000 validation samples. The adamW optimizer is employed with a learning rate of l_r_ = 10^−4^, a weight decay of 0.01, and a batch size of 512. Binary Cross-Entropy Loss (BCELoss) is used for model evaluation, where the raw logits are directly utilized for loss computation to avoid numerical instability and improve computational efficiency without explicitly applying the Sigmoid function.

Given the weaker performance of accumulated detection under extremely low SNR for non-fluctuating targets, the model is trained exclusively on non-fluctuating target scenarios. More specifically, the RD-Transformer is trained on non-fluctuating point-target echoes with additive white Gaussian receiver noise over a wide SNR range. During evaluation (Section 5), the trained model is directly applied, without any fine-tuning, to test sets that introduce Swerling I–IV amplitude-fluctuation statistics and a broader range of target kinematics using both Sinc-based and RTX-based echo synthesis. The fact that the detector maintains its SNR advantage across these unseen fluctuation models indicates that the reported gains do not rely on an exactly matched Swerling/noise configuration at training and test time but instead reflect robustness to this family of simulated distribution shifts. The resulting model is generalized across various Swerling cases to ensure robust detection in diverse signal environments.

## 5. Experiments and Results

We evaluated the performance of the RD-Transformer model in detecting extremely weak targets under noisy backgrounds, utilizing 10-pulse accumulation, and compared it against the state-of-the-art energy accumulation detection methods in radar systems. Experimental results demonstrate that the model consistently achieves target detection while satisfying the required false alarm constraints across all target fluctuation scenarios. Notably, this includes cases where the signal-to-noise ratio (SNR) of the received echoes is significantly lower than the critical SNR required by optimal energy accumulation detection to achieve a specified detection probability. To make the presentation of these results clearer, Section 5.2 provides qualitative detection examples at representative SNR values, Section 5.3 reports quantitative detection probability versus SNR curves under different Swerling fluctuation models, Section 5.4 analyzes false alarm behavior as a function of the detection gate, Section 5.5 presents ablation experiments isolating the contributions of cross-attention, dual encoders, and sliding-window preprocessing, and Section 5.6 summarizes the inference latency of small- and large-scale RD-Transformer models.

### 5.1. Data Preparation Details

The existing number of observed samples is too small compared to the scale of model parameters, making it difficult for the model to converge even with existing real experimental data samples. Therefore, we employ Sinc method data preparation and ray tracing method data preparation to achieve high-confidence generation of target-received echo signals under different scenarios and motion states. The Sinc method has low computational complexity but low fidelity, while the ray tracing method has high computational complexity but high fidelity. Both methods are used simultaneously in this experiment to achieve a balance between computational complexity, sample size, and sample quality.

#### 5.1.1. Parameter Settings

Basic parameters include radar transceiver-related parameters and target motion characteristic parameters. Among them, radar transceiver-related parameters include carrier frequency *Fc* = 1 GHz, sampling rate *Fs* = 10 MHz, baseband signal bandwidth *B* = 1 MHz, pulse width *PW* = 10 us, linear frequency modulation signal waveform, transmit-receive antenna gains (*Gtx* = *Grx* = 35 dB), equivalent noise temperature *T* = 293.5 K, load resistance of 50 Ω, noise factor of 3 dB, pulse repetition interval *PRI* = 100 μs and coherent accumulation pulse number *N* = 10. Target motion characteristic parameters include target position, velocity, acceleration, and jerk, with parameters set within observable distance and velocity ranges, and specific parameter values generated through uniform distribution random sampling during training. The parameters are detailed in Table 1.

#### 5.1.2. Sinc Method Data Preparation

The received echo signals obtained by radiating with different signals are generally subjected to pulse compression processing to form peaks, followed by subsequent accumulation processing. Therefore, our work refers to the relevant work [6] based on the point target model, using the Sinc function to achieve rapid generation of echoes in different scenarios. Considering the motion characteristics of the target, the construction method of target echo generation is as follows:

(15) $$y_{m}\left(t\right)\approx exp\left(-j\frac{4\pi F_{c}}{c}R_{ref}\right)\cdot exp\left(j\frac{4\pi F_{c}}{c}F\right)\cdot sinc\left(B\left(t-\frac{2}{c}\left(R_{ref}-F\right)\right)\right),$$

representing the radial motion of the target relative to the reference position, where $R_{ref}$ is the reference range for Taylor expansion, and F represents the radial displacement function defined in Equation (16). Due to the motion process, the function has high-order derivatives, so the Taylor series expansion around τ = 0 relative to the reference position is

(16) $$F=\sum_{\iota =1}^{k}\frac{R^{\left(l\right)}\left(0\right)}{\iota !}{\left(T_{st}m\right)}^{\iota }$$

In the current study, reference [61] and others have shown that using a third-order approximation can effectively reflect the motion characteristics of the target. Based on the third-order derivatives (k = 3), the target’s velocity, acceleration, and jerk are denoted as, respectively:

(17) $$F\left(v,a,\dot{a},T_{st},m\right)=v\cdot \left(T_{st}m\right)+\frac{1}{2}a\cdot {\left(T_{st}m\right)}^{2}+\frac{1}{6}\dot{a}\cdot {\left(T_{st}m\right)}^{3}$$

The fluctuation characteristics of the target RCS were modeled using the Swerling framework. Considering that slow fluctuations exhibit stability within a coherent pulse interval (CPI) but vary across scans (between different CPIs), the detection process focuses on accumulation within a single CPI. Consequently, scenarios involving slow-fluctuation targets (Swerling I and Swerling III) were consolidated for analysis.

#### 5.1.3. RTX Data Preparation

Although the point target model can generate a large number of sample data for training quickly, it cannot accurately model the target echoes in the scene, owing to ignoring factors such as multipath transmission, reflections from different target materials, and complex modulation effects of large targets (individual targets larger than one or multiple resolvable distance units) due to attitude motion, leading to differences between the generated data sample characteristics and actual measured data samples. We refer to the ray tracing method with NVIDIA^®^ OptiX™ 8.1.0 (NVIDIA Corporation, Santa Clara, CA, USA) for signal-level simulation of radar-received pulses [68]. In the above method, we no longer equate the received signal to the Sinc function but instead overlay the original transmitted signal x(t) after multi-path radiation attenuation. Each path calculates delay and Doppler according to the radar equation. Delay is obtained by integrating the motion of the target at the radiation-reflection time according to the target’s motion speed; Echo Doppler is calculated by sampling the target’s velocity at the moment of reflection.

### 5.2. Model Performance Outcomes

**Figure 4:**
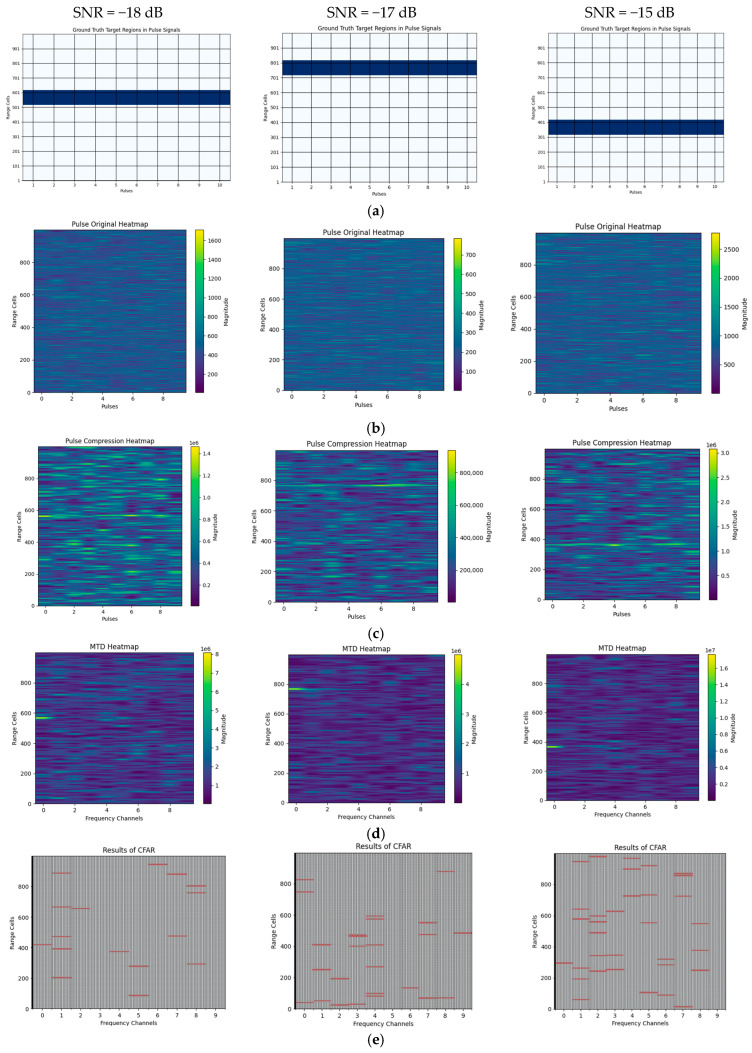

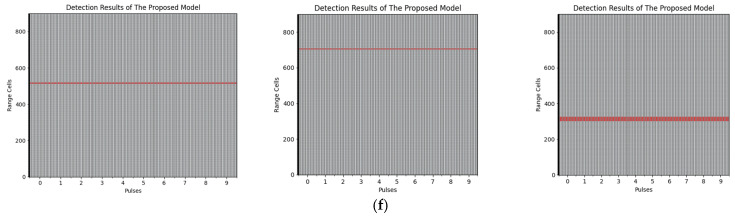
Comparison of detection results for extremely weak small targets in Swerling I/III scenarios using the traditional optimal energy detection method and the proposed model. (**a**) Ground truth position of the target within the pulse cell in the scene. The x-axis represents the slow time index, and the y-axis represents the fast-time range cell. (**b**) Raw received echo data (magnitude after IQ processing). (**c**) Results of the pulse compression. (**d**) Results of the coherent integration. (**e**) Results of CFAR in the time-Doppler domain. (**f**) Detection results of the proposed model, showing the target in fast-slow time.

We compare the performance of the proposed model against the optimal radar energy accumulation detection model under identical pulse accumulation conditions, as shown in Figure 4. For 10-pulse coherent accumulated echo sequences with SNR of −18 dB, −17 dB, and −15 dB, the proposed model successfully detects the target while maintaining control over false alarms. In contrast, conventional radar optimal energy detection methods, such as optimal pulse compression, range-Doppler coherent accumulation, and constant false alarm rate (CFAR) detection, struggle under these conditions. The extremely weak target echo signals result in inconspicuous target features across various conventional transform domains, leading to either excessive false alarms or an inability to detect any targets. In the conventional processing pipeline used for comparison, the complex I/Q echoes within each CPI are first matched-filtered using the known transmit waveform. The resulting outputs are then coherently integrated over N pulses in the range-Doppler domain based on the true PRI. Finally, detection is performed using a cell-averaging CFAR detector.

### 5.3. Coherent Detection Performance

**Figure 5:**
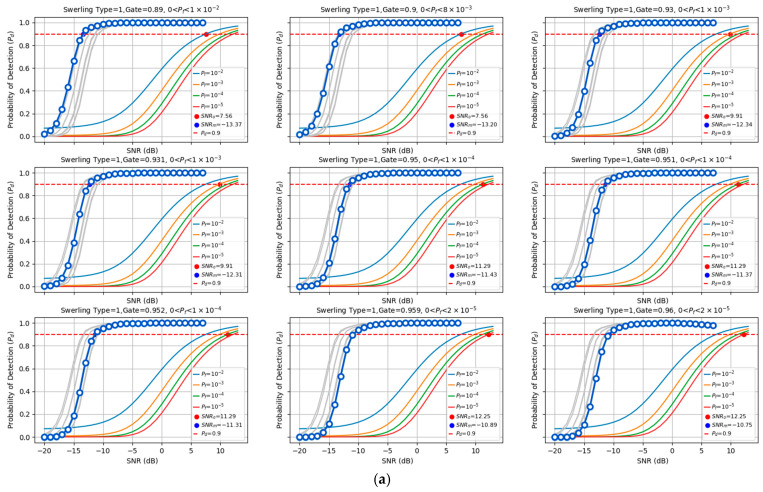

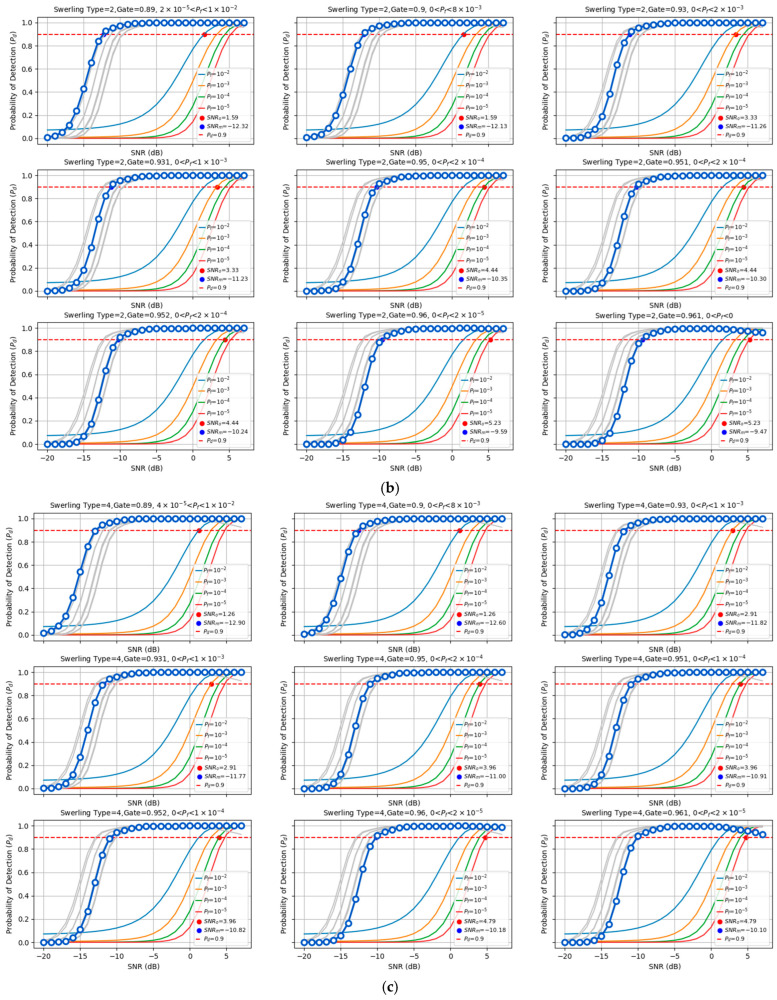
Detection performance comparison. (**a**) Detection performance comparison under Swerling I/III target fluctuation scenario with received SNR range of −20 to 10 dB and 10-pulse accumulation. (**b**) Detection performance comparison in Swerling II. The proposed method demonstrates a reduction of 13.72 dB to 14.83 dB in the required SNR to achieve a detection probability (P_d_) of 90%, compared to the traditional optimal energy accumulation detection method. (**c**) Detection performance comparison in Swerling IV. The proposed method demonstrates a reduction of 13.86 dB to 14.97 dB in the required SNR to achieve a detection probability (P_d_) of 90%, compared to the traditional optimal energy accumulation detection method.

We compared the detection performance of the proposed model against the optimal energy detection method employed in traditional radar systems under different target fluctuation scenarios (Swerling I/II/III/IV). The evaluation examined the effectiveness of low-pulse accumulation (coherent accumulation of 10 pulses), and the detection probabilities were analyzed under various false alarm control levels, as shown in Figure 5. The proposed method reduces the required SNR to achieve P_d_ = 90% by 20.76 dB to 23.14 dB compared to the optimal traditional energy accumulation method. For traditional radar, the optimal energy accumulation detection performance is numerically calculated using the incomplete gamma function [42]. The detection performance of the proposed model was statistically obtained through independent repeated experiments. In the experiments, each SNR point was evaluated with 105 repetitions to ensure statistical reliability.

### 5.4. False Alarm Rate

**Figure 6:**
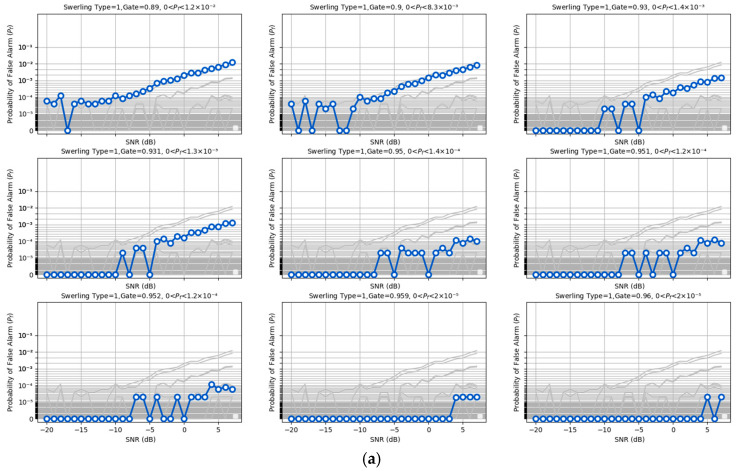

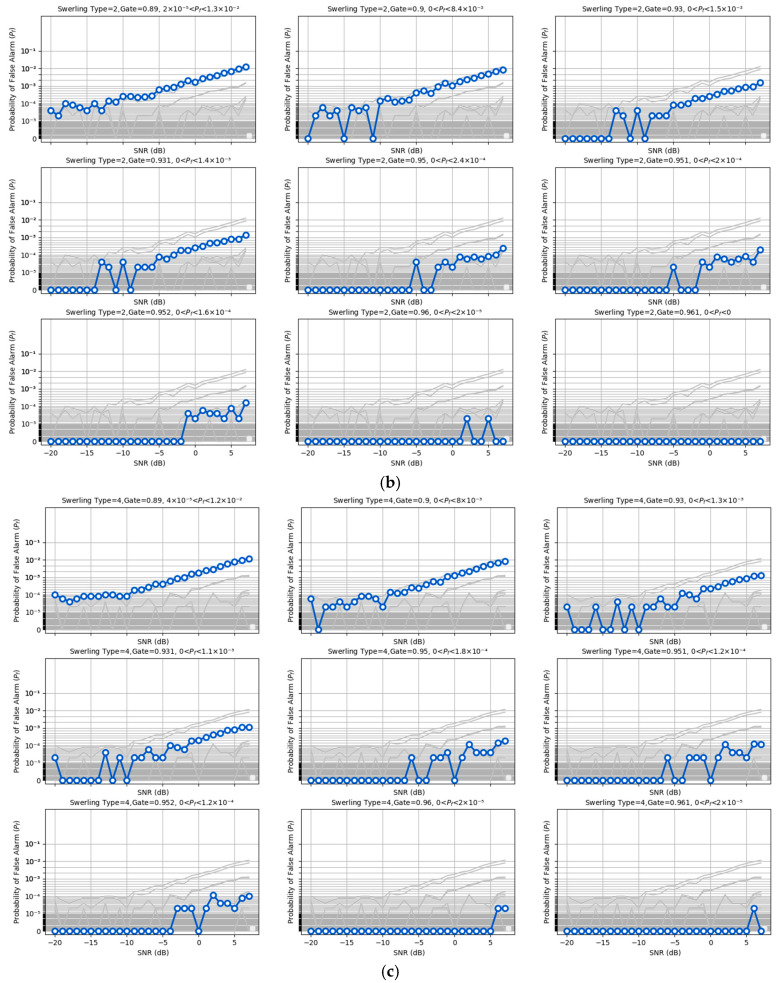
False alarm rate statistics. (**a**) False alarm rate statistics in Swerling I/III scenario. (**b**) False alarm rate statistics in Swerling II. The false alarm rate is controlled by adjusting the detection gate. As the SNR increases, the false alarm probability rises. In this study, the maximum false alarm probability is used to characterize the control performance under the current gate settings. (**c**) False alarm rate statistics in Swerling IV. The model’s false alarm rate in the Swerling IV scenario shows a slightly smaller increase compared to that in the Swerling II scenario.

To align with the statistical methodology of energy detection, the proposed model incorporates a mechanism for false alarm control. The model outputs are processed using a tunable sigmoid function and a gating mechanism, enabling flexible adjustments to achieve the desired false alarm performance. Specifically, the sigmoid scaling parameter sigmoid_k is set to 0.1, while the gate threshold is tuned within the range of 0.890 to 0.961. This configuration effectively constrains the model’s false alarm rate between 10^−2^ and 10^−5^, ensuring comparability with optimal energy detection methods. The model’s False alarm rate exhibits slight variations with changes in the received SNR. These variations were characterized through independent repeated experiments, and the results are presented in Figure 6 Panels 1–9 (arranged from top left to bottom right) correspond to different False alarm rate levels, panels 1 and 2 represent 10^−2^ false alarm level, panels 3 and 4 represent 10^−3^ false alarm level, panels 5, 6, and 7 represent 10^−4^ false alarm level, and panels 8 and 9 represent 10^−5^ false alarm level. It is important to emphasize that, for all comparative experiments in this study, the maximum false alarm rate observed under controlled conditions was used as the baseline for evaluating detection. Notably, across most SNR ranges, the model’s false alarm rate remained below the constant false alarm rate. From this perspective, the proposed model demonstrates superior performance compared to conventional energy detection methods. More specifically, for each pair of sigmoid_k and gate thresholds, the false alarm probability was estimated via large-scale Monte Carlo trials over a wide low-SNR range covering the operating conditions in Figure 6, and the reported $P_{f}$ corresponds to the maximum value observed across all SNR points in this range. Hence, for any SNR within this interval, the actual false alarm probability of the proposed detector does not exceed the $P_{f}$ level. We also note that the present study evaluates $P_{f}$ under the simulated thermal noise models described in Section 5.1; in practical deployments with more complex non-Gaussian noise or clutter, the same k-sigmoid gating can be re-calibrated offline using a small amount of site-specific data to restore the desired false alarm level, while a systematic investigation of such scenarios is left for future work.

### 5.5. Ablation Experiments

To systematically validate the individual contributions of the three key design components in the proposed method—namely, the cross-attention fusion mechanism, the dual-encoder architecture, and the sliding-window preprocessing strategy—this section presents a comprehensive ablation study. All ablation experiments adhere to a unified evaluation protocol to ensure fair comparison. The training hyperparameters, optimizer configuration, and early-stopping criteria remain identical to those employed in the main experiments (Section 4). Synthetic data generation follows the procedure detailed in Section 5.1. For each SNR operating point, 1 × 10^6^ independent trials are conducted on separate test sets to ensure statistical reliability. The detection performance is evaluated across SNR ∈ [−20, 10] dB with 0.1 dB increments, yielding probability of detection (Pd) versus SNR curves under a fixed false alarm rate P_f_ = 1 × 10^−4^. The primary performance metric is the critical SNR required to achieve P_d_ = 90%, denoted as SNR_0_, which quantifies the detection threshold under operationally relevant conditions.

#### 5.5.1. Ablation 1: Cross-Attention Versus Simple Fusion Strategies

This ablation study investigates whether the cross-attention mechanism provides significant advantages over commonly used simple fusion strategies, thereby validating its role in extracting Tx-Rx mutual information and enhancing detection sensitivity at low SNR. Three fusion strategies are compared under otherwise identical configurations:

(1.1) Cross-attention (Proposed): Dual-encoder + Cross-attention decoder + Sliding-window preprocessing.
(1.2) Concat + FC (Variant A): The received and transmitted feature sequences are concatenated along the feature dimension, followed by two fully connected (FC) layers for fusion. All other components remain unchanged.
(1.3) Dot-product + FC (Variant B): The received and transmitted features undergo channel-wise inner product, followed by FC layers for dimensionality reduction and fusion. All other components remain unchanged.

The results in Table 2 demonstrate that cross-attention achieves an SNR_0_ improvement of 1.10 dB relative to concatenation-based fusion and 1.50 dB relative to dot-product fusion. The superior performance of cross-attention can be attributed to its ability to dynamically align received and transmitted features at each pulse/time position through learned attention weights, thereby selectively enhancing weak-energy channels that are critical for target detection. This adaptive alignment is particularly beneficial in low-SNR regimes. In contrast, simple concatenation or dot-product fusion treats all feature channels uniformly, failing to capture the fine-grained temporal correspondence between transmitted waveforms and received echoes. In accordance with the Monte Carlo evaluation protocol described at the beginning of Section 5.5, the P_d_-SNR curves from which SNR_0_ is extracted are obtained from large numbers of independent trials at each SNR operating point.

#### 5.5.2. Ablation 2: Dual-Encoder Versus Shared-Encoder Architecture

This experiment examines whether processing the transmitted (Tx) and received (Rx) pathways with dedicated encoders preserves pathway-specific characteristics and improves cross-pathway mutual information modeling.

Two architectural variants are evaluated:

(2.1) Dual-encoder (Proposed): Dual-encoder with independent parameters for Tx and Rx pathways + Cross-attention fusion + Sliding-window preprocessing.
(2.2) Shared-encoder (Variant C): A single encoder with shared weights processes both Tx and Rx inputs sequentially. The cross-attention mechanism remains unchanged, operating on the encoded representations, see Table 3.

The dual-encoder architecture achieves an SNR_0_ improvement of 0.80 dB compared to the shared-encoder configuration. The advantage of the dual-encoder design stems from its capacity to learn pathway-specific inductive biases: the Tx encoder can specialize in extracting fine-grained features of the transmitted reference signal (e.g., pulse shape, modulation characteristics), while the Rx encoder focuses on capturing received signal characteristics under noise and clutter interference. These specialized representations facilitate more precise alignment during the cross-attention stage, enabling the decoder to better exploit the mutual information between transmitted waveforms and received echoes. In contrast, a shared encoder must learn a generic representation that accommodates both pathways, potentially diluting pathway-specific features critical for weak target detection.

#### 5.5.3. Ablation 3: Sliding-Window Versus Patch-Based Preprocessing

Two preprocessing strategies are compared:

(3.1) Sliding-window (Proposed): Sliding-window with parameters specified in Section 4.1 (window length and stride) + Dual-encoder + Cross-attention.
(3.2) Patch-based (Variant D): The fast-time signal stream is partitioned into non-overlapping or minimally overlapping patches. The number of tokens is kept comparable to the baseline to ensure a fair comparison of representational capacity.

The sliding-window approach yields an SNR_0_ improvement of approximately 0.70 dB relative to patch-based partitioning. This advantage is particularly pronounced under challenging conditions involving low SNR and limited pulse counts, suggesting that preserving fast-time local continuity plays a critical role in coherent energy integration and temporal consistency modeling for weak targets. Patch-based partitioning, while conceptually simpler and more compatible with image-based processing pipelines, may inadvertently disrupt the temporal correlation structure essential for detecting faint echoes embedded in noise. The boundary discontinuities introduced by non-overlapping patches can fragment coherent signal components across adjacent patches, degrading the model’s ability to aggregate energy across multiple range bins, see Table 4.

#### 5.5.4. Summary of Ablation Results

The ablation studies collectively validate the design rationale of the proposed method. The cross-attention mechanism contributes the largest performance gain (1.1–1.5 dB), demonstrating its critical role in extracting cross-pathway mutual information. The dual-encoder architecture provides a moderate but meaningful improvement (0.8 dB) by preserving pathway-specific features. The sliding-window preprocessing strategy offers an additional 0.7 dB gain by maintaining temporal continuity. Together, these components form a synergistic system optimized for coherent weak target detection in pulse-Doppler radar systems.

### 5.6. Inference Speed

To meet real-time processing requirements, the GPU coherent processing time (ProcessTime) for N pulses in a single range unit must be less than *N* × *CPI*. Here, ProcessTime denotes the average wall-clock latency from ingesting the complex I/Q samples of one coherent processing interval (CPI) for a single range unit, through sliding-window preprocessing, embedding, dual-encoder and cross-attention inference, k-sigmoid gating, and threshold comparison, to outputting the final binary detection result. In other words, the reported figures correspond to the full online processing chain from I/Q input to detection output, rather than only the bare neural-network forward pass. Inference latency for models of different scales is evaluated, as shown in Table 5. The small-scale model, tested on a platform equipped with an NVIDIA 3080Ti GPU (NVIDIA Corporation, Santa Clara, CA, USA), achieved a processing time of 4.60 × 10^−5^ s. The large-scale model, evaluated on a platform with two NVIDIA Tesla V100 GPUs, achieved a processing time of 1.08 × 10^−4^ s. With *N* × *CPI* = 1.00 × 10^−3^ s in this experiment, both models satisfy the real-time processing requirements. These values correspond to a per-range-cell inference latency of the small-scale model and large-scale model on the tested GPU platforms, only 4.6% and 10.8%, respectively, of the available coherent processing interval in this experiment. Thus, although RD-Transformer requires more floating-point operations than a traditional processing chain, its measured runtime remains well within real-time constraints for the radar parameters considered in this work.

To ensure real-time performance for fast-time detection, the required number of parallel GPUs must exceed ceil (ProcessTime × DisUnit/CPI). However, increasing the number of parallel GPUs does not always lead to better performance due to the overhead introduced by data access between GPUs and memory. Optimal configurations are achieved when ProcessTime × m_DisUnit/CPI approaches but remains below 1, providing a cost-effective balance between processing efficiency and computational resources. In practical deployments, different fast-time range units within a CPI are mutually independent and can be treated as separate samples, which are either batched on a single GPU or assigned to different GPUs without inter-GPU communication. When multiple GPUs are available, distributing disjoint groups of range units across devices avoids splitting a single range unit over several GPUs and thus mitigates performance degradation due to inter-GPU data transfers, leading to near-linear throughput scaling with the number of GPUs in typical deployment scenarios.

## 6. Conclusions

In this paper, we proposed RD-Transformer, an attention-based framework that achieves significant SNR reduction (14–20 dB) for weak target detection in pulsed radar systems by learning adaptive coherent integration from multi-pulse data. Key contributions include the following: (1) a cross-attention mechanism for transmit-receive feature fusion that implicitly compensates for target motion, (2) dual-encoder architecture preserving pathway-specific characteristics, and (3) a tunable false alarm control mechanism via k-sigmoid gating enables the same trained network to satisfy different P_d_–P_f_ required by radar systems without retraining.

Experimental results demonstrate substantial performance gains over optimal energy detection. However, two main limitations warrant future investigation: (1) all experiments rely on simulated data—validation on real radar measurements is essential to confirm practical effectiveness; (2) computational complexity may limit deployment on resource-constrained platforms. Future work will prioritize real-data validation, model compression (knowledge distillation, pruning), and extension to joint detection and motion parameter estimation tasks.

## Figures and Tables

**Table 1:** Transmitter and receiver parameters and target motion settings used in the experiments.

**Transceiver Parameters**					
**No.**	**Parameters**	**Parameter Setting**	**No.**	**Parameters**	**Parameter Setting**
1	Carrier Frequency (*Fc*)	1 GHz	7	Number of Accumulated Pulses	10
2	Sampling Rate (*Fs*)	10 MHz	8	Transmit Antenna Gain (*Gtx*)	35 dB
3	Baseband Signal Bandwidth (*B*)	1 MHz	9	Receive Antenna Gain (*Grx*)	35 dB
4	Pulse Width (*PW*)	10 us	10	Noise Temperature (*T*)	293.5 K
5	Signal Form	Linear Frequency Modulation (LFM)	11	Load Resistance	50 Ω
6	Pulse Repetition Interval (*PRI*)	100 us	12	Noise Figure	3 dB
**Target Motion Parameters**					
**No.**	**Parameters**	**Parameter Setting**	**No.**	**Parameters**	**Parameter Setting**
1	Initial Position (Dis)	U(5, 15) km	3	Acceleration (a)	U(0, 50) m/s^2^
2	Initial Velocity (v)	U(10, 500) m/s	4	Jerk	U(0, 5) m/s^3^

**Table 2:** Comparison of fusion mechanisms.

Method	SNR_0_ @ P_d_ = 90%, P_f_ = 1 × 10^−4^ (dB), Swerling I/III
Cross-attention (Baseline)	−11.4 dB
Concat + FC (Variant A)	−10.3 dB
Dot-product + FC (Variant B)	−9.9 dB

**Table 3:** Comparison of encoder architectures.

Method	SNR_0_ @ P_d_ = 90%, P_f_ = 1 × 10^−4^ (dB)
Dual-encoder (Proposed)	−11.4 dB
Shared-encoder (Variant C)	−10.6 dB

**Table 4:** Comparison of preprocessing strategies.

Method	SNR_0_ @ P_d_ = 90%, P_f_ = 1 × 10^−4^ (dB)
Sliding-window (Proposed)	−11.4 dB
Patch-based (Variant D)	−10.7 dB

**Table 5:** Inference speed statistics.

**Small-Scale Model**			
Number of Model Parameters		Model Storage Space	
2.85 × 10^6^		16.57 MB	
Platform	Sample Count	Processing Delay	Single Sample Processing Delay
NVIDIA 3080Ti × 1	10^5^	4.60 s	4.60 × 10^−5^ s
**Large-Scale Model**			
Number of Model Parameters		Model Storage Space	
6.50 × 10^7^		300.84 MB	
Platform	Sample Count	Processing Delay	Single Sample Processing Delay
NVIDIA Tesla V100 × 2	10^5^	10.81 s	1.08 × 10^−4^ s

## Data Availability

The original contributions presented in this study are included in the article. Further inquiries can be directed to the corresponding author.
